# Dissolution Profile of Tosufloxacin Tosylate in Biorelevant Bicarbonate Buffer Containing Sodium Chloride: Precipitation of Hemi-hydrochloride Salt at the Particle Surface

**DOI:** 10.1007/s11095-025-03905-4

**Published:** 2025-08-11

**Authors:** Nanami Okamoto, Hibiki Yamamoto, Kiyohiko Sugano

**Affiliations:** Molecular Pharmaceutics Lab, College of Pharmaceutical Sciences, Ritsumeikan University, 1-1-1, Noji-Higashi, Kusatsu, Shiga 525-8577 Japan

**Keywords:** Biorelevant, Chloride ion, Dissolution, Particle surface, Salt

## Abstract

**Purpose:**

The purpose of the present study was to investigate the dissolution profile of tosufloxacin tosylate monohydrate (TFLX TS) in biorelevant bicarbonate buffer (BCB). TFLX is a zwitterionic drug (p*K*_*a*_: 5.8 and 8.7), formulated as tosylate salt to enhance its dissolution.

**Methods:**

The dissolution profiles were measured in BCB or phosphate buffer (PPB) at pH 6.5 with buffer capacity (β) = 0.88 or 4.4 mM/pH, containing NaCl or Na_2_SO_4_ (*I* = 0.14 M). The residual particles were analyzed by powder X-ray diffraction. In a separate study, the particle surface of TFLX TS after contact with BCB was observed by scanning electron microscopy and real-time polarized light microscopy. The pH solubility profile of TFLX was measured using HCl and TS solutions.

**Results:**

TFLX TS dissolved less in the NaCl media than in the Na_2_SO_4_ media. It also dissolved less in PPB than in BCB (at β = 4.4 mM/pH), and less at β = 4.4 mM/pH than at 0.88 mM/pH. The analysis of residual particles indicated that the hemi-hydrochloride salt (TFLX 1/2HCl) precipitated on the particle surface of TFLX TS in the NaCl media. In contrast, the free form of TFLX precipitated in the Na_2_SO_4_ media. The pH solubility profile matched the hemi-hydrochloride stoichiometry (*K*_*sp*_ = [TFLX∙H^+^][TFLX][Cl^−^]).

**Conclusion:**

The dissolution of TFLX TS in the NaCl media was suppressed by the precipitation of TFLX 1/2HCl on the particle surface. This is an important case showing that NaCl can suppress the dissolution profile of a non-HCl drug salt at a neutral pH.

**Supplementary Information:**

The online version contains supplementary material available at 10.1007/s11095-025-03905-4.

## Introduction

Dissolution tests have been widely used to assess the oral absorption of a drug. Biorelevant dissolution test media have been used to evaluate the dissolution, supersaturation, and precipitation profiles of a drug (hereinafter referred to as the dissolution profile) in the gastrointestinal tract [1, 2]. In the small intestine, the pH value is maintained by bicarbonate buffer (BCB) [3]. BCB undergoes the chemical equilibrium of HCO_3_^−^ + H^+^  ⇌ H_2_CO_3_ ⇌ H_2_O + CO_2_ [4]. The reaction rate of CO_2_ hydration is significantly slower than that of H_2_CO_3_ dehydration. This unique property of BCB affects the dissolution profile of ionizable drugs and excipients [5–11]. However, BCB has not been widely used because of practical difficulties [12]. Instead, phosphate buffer solutions (PPB) have been widely used as dissolution media. Recently, the floating lid method was developed as a simple and versatile method to use BCB [13–18]. This method does not require CO_2_ bubbling, thus avoiding the formation of surfactant foam and the artifact stimulation of crystal precipitation [19]. In addition, sodium chloride (NaCl) is typically used to adjust the osmolarity and ionic strength (*I*) of biorelevant media [1, 2]. The osmolarity and *I* of a fluid would be important for the assessment of controlled-release formulations [20]. However, the impact of NaCl on the dissolution profile of drug substances has been little investigated.

**Fig. 1:**
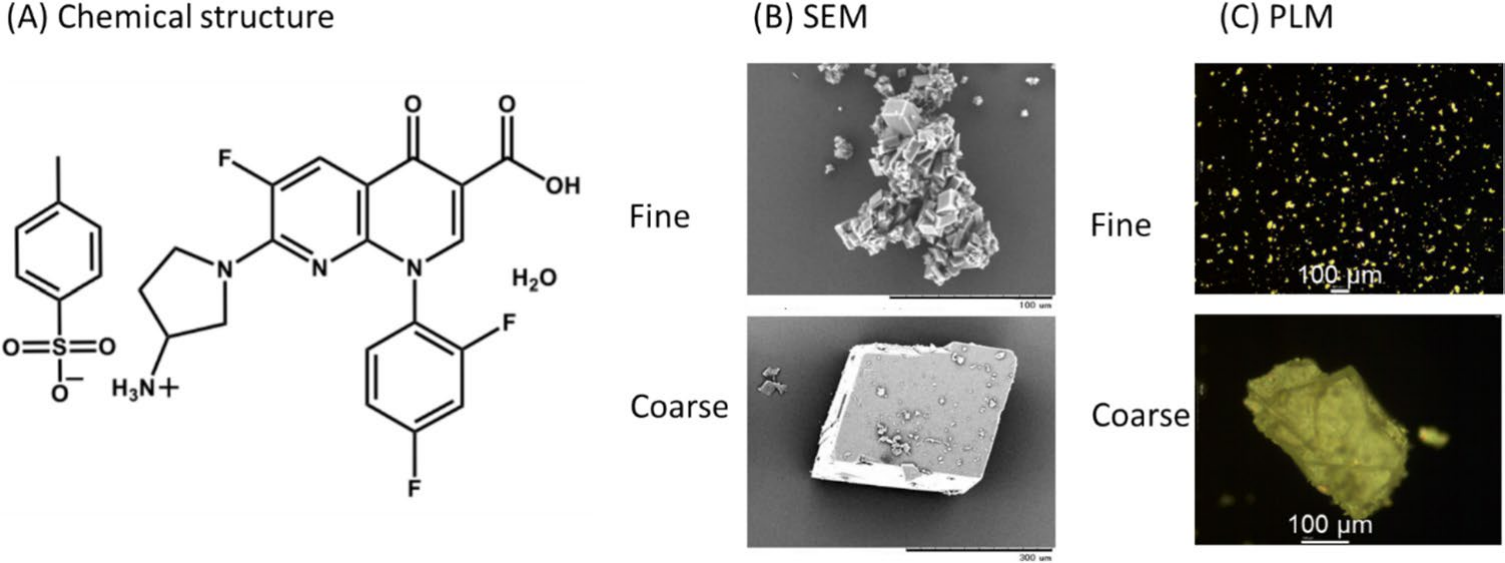
Chemical structure of tosufloxacin tosylate monohydrate (TFLX TS) (**A**), the scanning electron microscope image (SEM) (**B**), and the polarized light microscope (PLM) image (**C**) of initial TFLX TS particles. Fine and coarse particles were prepared by recrystallization from an ethanol–water mixture (see text). The samples for PLM were dispersed in silicone oil.

Tosufloxacin tosylate monohydrate (TFLX TS) shows incomplete bioavailability in humans [21]. TFLX is a zwitterionic drug with p*K*_*a1*_ = 5.8 and p*K*_*a2*_ = 8.7 (Fig. 1) [21]. TFLX shows poor equilibrium solubility (*S*_*eq*_) at pH 6.5 (*S*_*eq*_ = 2.1 μg/mL at 37°C) [22]. Therefore, TFLX is formulated as a salt with a strong acid (p-toluenesulfonic acid), and L-aspartic acid was added as an acidifying excipient in the commercial tablet. However, in our previous study, the TFLX TS tablet still showed poor dissolution profiles in the BCB-based fasted state simulated intestinal fluid (BCB-FaSSIF) at pH 6.5 [23, 24]. In those studies, the dissolution profiles of the TFLX TS tablet in BCB differed from those in PPB. Because the equilibrium solubility of TFLX was increased by bile micelles in FaSSIF [24], the other component of biorelevant media would have suppressed the dissolution process of TFLX TS.

The purpose of this study was to investigate the dissolution profile of TFLX TS drug substance in biorelevant BCB in detail. This study used BCB that reflects the intestinal environment for dissolution tests (pH 6.5, 10 mM (buffer capacity (β) = 4.4 mM/pH), containing NaCl (*I* = 0.14 M)). For comparison, PPBs with the same β and *I* were used. To examine the effect of NaCl while maintaining *I*, Na_2_SO_4_ was used to adjust *I* instead of NaCl. Na_2_SO_4_ was selected because it is composed of a non-halogen divalent anion (SO_4_^2−^) that is considered to behave differently from Cl^−^. In addition, since a decrease in β has been shown to increase the dissolution of a salt-form drug [25], the dissolution profiles at a lower β value were also investigated (β = 0.88 mM/pH).

## Materials and Methods

### Materials

Tosufloxacin tosylate monohydrate (TFLX TS), p-toluenesulfonic acid monohydrate, NaH_2_PO_4_∙2H_2_O, NaHCO_3_, NaCl, Na_2_SO_4_, 8 mol/L NaOH, and 6 mol/L HCl were purchased from FUJIFILM Wako Pure Chemical Corporation (Osaka, Japan). Ethanol was purchased from Maruishi Pharmaceutical Co., Ltd. (Osaka, Japan).

### Methods

#### Preparation of Sample and Reference Materials

Tosufloxacin tosylate monohydrate (TFLX TS) was recrystallized before use for dissolution tests. TFLX TS obtained from the supplier (3.41 mmol) was added to an ethanol–water mixture (1:1 (v:v), 40 mL) in a conical beaker. A small excess amount of p-toluenesulfonic acid (0.341 mmol) was added to shift the equilibrium to the salt form. TFLX TS was completely dissolved by heating to 70°C. The solution was stirred using a magnetic stirrer for 6 h at room temperature (25 ± 2°C) to obtain fine particles. Coarse particles were obtained by slowly cooling the solution to room temperature in a styrofoam container for 12 h without stirring. The precipitated crystals were harvested by vacuum filtration and analyzed by powder X-ray diffraction (PXRD) (Supplementary Material Figure S1). The polarized light microscope image analysis indicated that the average sizes of fine and coarse particles were about 11 and 217 μm, respectively (Fig. 1C) [26].

The free form of TFLX (TFLX FF) was prepared as follows. TFLX TS (9.82 mmol) was completely dissolved in an ethanol–water mixture (1:1 (v/v), 400 mL) at 70°C. An equimolar amount of NaOH (1 M NaOH, 9.82 mL) was added to induce the precipitation of TFLX FF. The suspension was left to stand for 12 h at room temperature without stirring. The precipitated crystals were collected by vacuum filtration and analyzed by PXRD and thermogravimetry/differential thermal analysis (TG/DTA). No PXRD peak of TFLX TS was observed (Supplementary Material Figure S1). The TG/DTA data suggested that it includes about 2 H_2_O per TFLX after vacuum filtration (Supplementary Material Figure S2). After storing under 75% relative humidity (RH) for 3 months at room temperature, the sample contained about 2.2 H_2_O. After storing under 100% RH for 3 months, the sample contained about 3.7 H_2_O (measured immediately after removal from the chamber), suggesting that it can form a higher number of hydrates in aqueous media. However, it rapidly returned to about 2H_2_O during handling at ambient humidity. Therefore, this material was referred to as TFLX FF in this report.

The hydrochloride salt of TFLX was prepared as follows. TFLX FF (0.21 mmol) was added to distilled water (500 mL) and completely dissolved by the addition of NaOH (1.0 M, 0.42 mL) at 70°C. HCl (1.0 M, 0.63 mL) was added to induce the precipitation of an HCl salt. The suspension was left to stand for 12 h at room temperature without stirring. The precipitated crystals were analyzed by PXRD and TG/DTA. No PXRD peak of TFLX FF was observed (Supplementary Material Figure S1). The pH titration measurement suggested the formation of a hemi-hydrochloride (TFLX 1/2HCl) composed of one protonated TFLX (TFLXH^+^), one free-form TFLX, and one chloride ion (Cl^−^) (Supplementary Material Figure S3). The solubility product (*K*_*sp*_) is defined as *K*_*sp*_ = [TFLXH^+^][TFLX][Cl^−^]. The pH solubility profile measured by adjusting the pH using HCl also supported this stoichiometry (discussed later). The TG/DTA data indicated that it includes 0.54 H_2_O per TFLX (Supplementary Material Figure S4). After storing under 75% RH for 2 months at room temperature, the sample contained about 0.74 H_2_O. After storing under 100% RH for 3 months, the sample contained about 10.0 H_2_O (measured immediately after removal from the chamber), suggesting it can form a higher number of hydrates in aqueous media. Similar to the case of TFLX FF, it rapidly returned to about 0.5 H_2_O during handling at ambient humidity. Therefore, this material was referred to as TFLX 1/2HCl in this report.

Single-crystal X-ray structure analysis is ideal for confirming the crystal form and stoichiometry. However, no suitable crystals were available for either TFLX FF or TFLX 1/2HCl.

#### Dissolution Test

A mini-paddle dissolution apparatus (NTR-6200A; Toyama Sangyo Co., Ltd., Osaka, Japan) was used for the dissolution test. A NaHCO_3_ solution (98 mL, 2.04 mM or 10.2 mM), prewarmed at 37°C in a container for at least 30 min, was added to each vessel. The temperature was maintained at 37°C. The paddle rotation speed was set to 100 rpm. An HCl solution (2.0 mL) (0.033, or 0.165 M) was added to adjust the pH value to pH 6.5 (the total volume was 100 mL). These HCl concentrations were experimentally pre-determined to give pH 6.5 after adding to the NaHCO_3_ solutions (BCB 2.0 or 10 mM (β = 0.88 or 4.4 mM/pH), *I* = 0.14 M adjusted by NaCl or Na_2_SO_4_) (Table I). The solution surface was covered with a floating lid for a mini-vessel [27]. The conditions of PPB were the same as BCB buffer, including the use of the floating lid (pH 6.5, 1.6 or 8.0 mM (β = 0.88 or 4.4 mM/pH), *I* = 0.14 M adjusted by NaCl or Na_2_SO_4_) (Table I). TFLX TS (30 mg, fine particles) was added to each vessel. At specified time intervals, a small volume of samples (0.5 ml) was withdrawn and immediately filtered (hydrophilic PVDF, φ = 4.0 mm, pore size: 0.22 µm, Merck). The first few droplets were discarded to avoid filter adsorption. The filtrate was diluted with an appropriate medium, and the concentration of TFLX was measured by UV absorbance at 365 nm (SH-9500lab, CORONA ELECTRIC, Ibaraki, Japan). The final pH value was measured using a pH meter (300-P–C, HORIBA Advanced Techno, Co., Ltd., Kyoto, Japan). The residual particles were collected by vacuum filtration and analyzed by PXRD.

The β values were calculated by the Van Slyke equation [28] using the p*K*_*a*_ values close to pH 6.5 (BCB: p*K*_*a*_ = 6.05 (for *I* = 0.15 M, at 37°C), PPB: p*K*_*a*_ = 6.69 (for *I* = 0.15 M, at 37°C)), assuming the activity coefficient of H^+^ is 1 [28].

#### Scanning Electron Microscopy of TFLX TS Particles Suspended in Bicarbonate Buffer

The coarse crystalline particles of TFLX TS were used for this experiment to observe the crystalline surface. TFLX TS was suspended in pH 6.5 10 mM BCB, *I* = 0.14 M adjusted by NaCl or Na_2_SO_4_, for 0.5, 3, and 5 min at room temperature. The crystals were collected by vacuum filtration. The crystal surfaces were observed by scanning electron microscopy (SEM, TM-1000, Hitachi High-Technologies Corporation, Tokyo, Japan).

#### Real-time Polarized Light Microscope Observation of TFLX TS Particles in Bicarbonate Buffer

The experimental procedures of real-time PLM observation have been previously reported in detail. In brief, the coarse crystalline particles of TFLX TS were placed on a glass slide and covered with another glass slide. These two glass slides were tightly pinched by using two binder clips to fix the particles between the glass slides. BCB was penetrated from the side of the glass slide at room temperature (30 μL, 10 mM BCB, pH 6.5, *I* = 0.14 M adjusted by NaCl or Na_2_SO_4_).

#### pH Solubility Profile Measurement

TFLX FF (8 to 20 mg) was added to an HCl or p-toluenesulfonic acid solution (5 to 10 mL) in a 15 mL test tube. The test tube was placed horizontally in a reciprocal shaker. After shaking for 48 h at 37 °C (at 90 rpm, motion width: 2.5 cm), the sample was filtered (hydrophilic PVDF, φ = 4.0 mm, pore size: 0.22 µm, Merck). The first few droplets were discarded to avoid filter adsorption. The concentration of TFLX was measured as described above. After measuring the final pH (9615S-10D Standard ToupH electrode (HORIBA Advanced Techno Co., Ltd., Kyoto, Japan)), the residual particles were collected by vacuum filtration and analyzed by PXRD.

#### Powder X-ray Diffraction

A sample was placed on a zero-diffraction plate and analyzed by PXRD (Rigaku Ultima IV, Rigaku Corporation, Tokyo, Japan). Data was collected from 5 to 35° (2θ) at a step size of 0.02° and scanning speed of 10 deg/min with Cu Kα radiation generated at 40 mA and 40 kV.

#### Thermogravimetric Analysis/Differential Thermal Analysis

The sample was placed in an aluminum pan (non-sealed) and analyzed by TG/DTA at 10°C/min under nitrogen gas (DTG-60AH, Shimazu Corporation, Kyoto, Japan).

**Table I:** Compositions of Dissolution Media

Buffer	β (mM/pH)	NaHCO_3_(mM)	NaH_2_PO_4_∙2H_2_O (mM)	HCl^a^ (mM)	NaOH^a^ (mM)	NaCl (mM)	Na_2_SO_4_ (mM)
BCB	0.88	2.0	-	0.62	-	-	46
	0.88	2.0	-	0.62		138	-
	4.4	10	-	3.3	-	-	44
	4.4	10	-	3.3		131	-
PPB	0.88	-	1.6	-	0.57	-	46
	0.88	-	1.6	-	0.57	137	-
	4.4	-	8.0	-	3.1	-	42
	4.4	-	8.0	-	3.1	126	-

a These concentrations were experimentally pre-determined to give pH 6.5 after adding to the NaHCO_3_ and NaH_2_PO_4_ solutions

## Results

### Dissolution Profiles of Tosufloxacin Tosylate Monohydrate

**Fig. 2:**
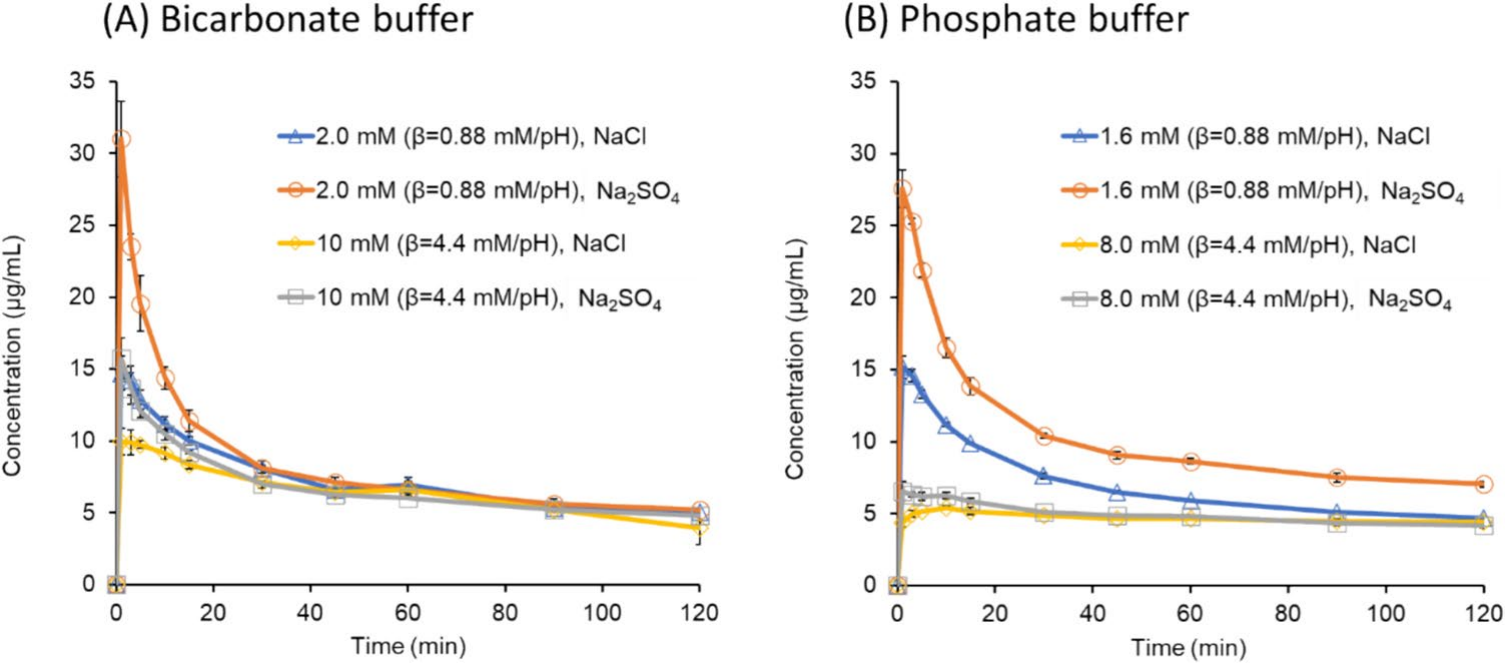
Dissolution profiles of TFLX TS in (**A**) bicarbonate buffer (BCB) and (**B**) phosphate buffer (PPB). Mean ± S.D., N = 3.

The dissolution profiles of TFLX TS are shown in Fig. 2. TFLX TS dissolved less in the NaCl media than in the Na_2_SO_4_ media. It also dissolved less in PPB than in BCB (at β = 4.4 mM/pH), and less at β = 4.4 mM/pH than at 0.88 mM/pH. The maximum TFLX concentration was less than 32 μg/mL in all cases. The final pH values are shown in Table II. When β = 4.4 mM/pH (10 mM BCB and 8.0 mM PPB), the final pH remained close to the initial pH (pH 6.5). When β = 0.88 mM/pH (2.0 mM BCB and 1.6 mM PPB), the final pH value was slightly reduced. The pH reduction in the Na_2_SO_4_ media was greater than that in the NaCl media.

**Table II:** Final pH Values After Dissolution Test with Initial pH Value of 6.5

Buffer species	Buffer concentration (mM)^a^	Ionic strength adjustment^b^	Final pH^c^
BCB	2.0 (β = 0.88 mM/pH)	NaCl	6.44 ± 0.07
		Na_2_SO_4_	6.19 ± 0.03
	10 (β = 4.4 mM/pH)	NaCl	6.52 ± 0.02
		Na_2_SO_4_	6.62 ± 0.11
PPB	1.6 (β = 0.88 mM/pH)	NaCl	6.13 ± 0.01
		Na_2_SO_4_	5.82 ± 0.04
	8.0 (β = 4.4 mM/pH)	NaCl	6.40 ± 0.02
		Na_2_SO_4_	6.45 ± 0.01

a Data in parentheses indicate buffer capacity (β)

b *I* = 0.14 M

c Mean ± S.D., N = 3

**Fig. 3:**
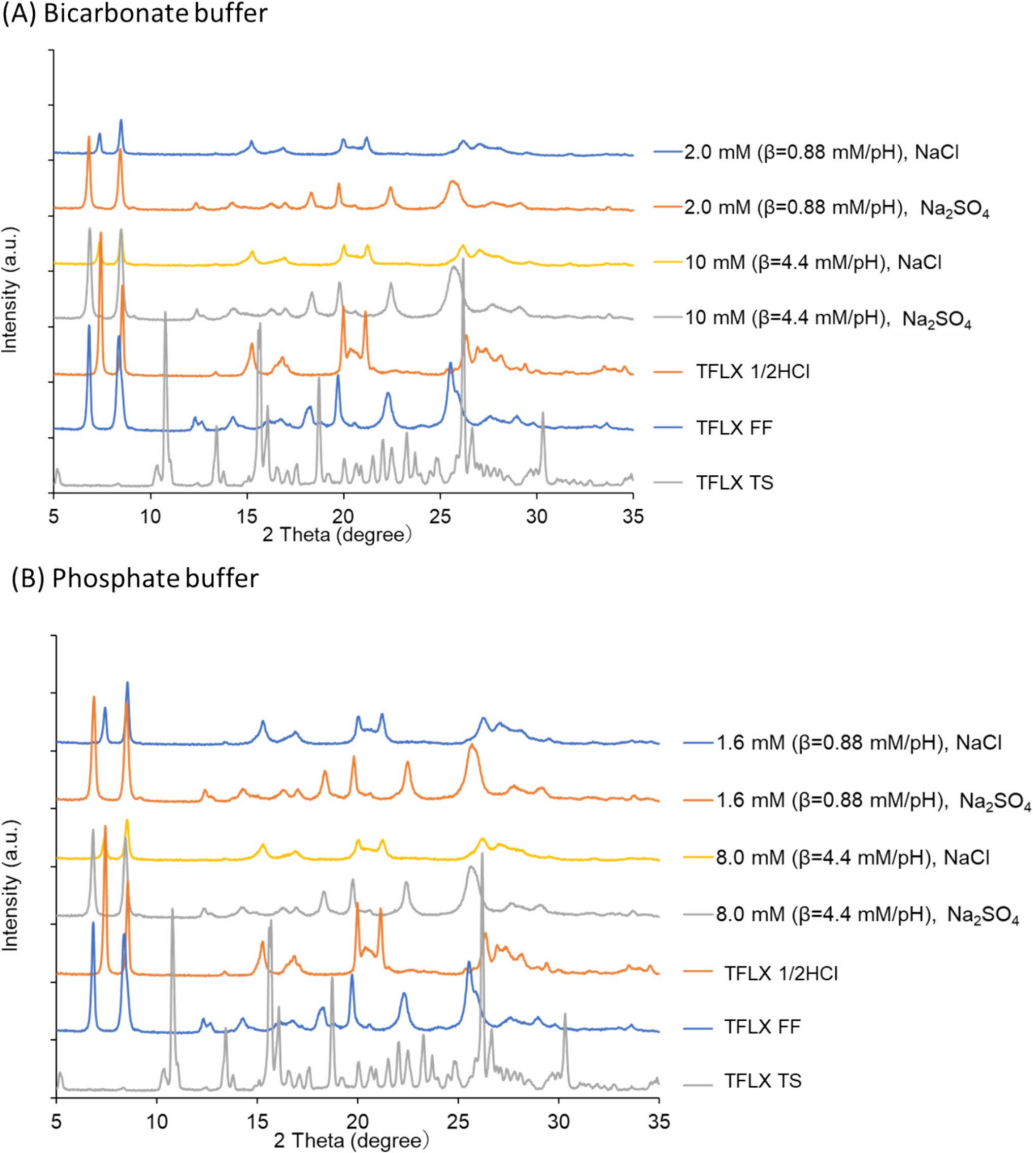
PXRD patterns of residual particles in dissolution tests after 2 h in (**A**) bicarbonate buffer (BCB) and (**B**) phosphate buffer (PPB).

The PXRD data of residual particles are shown in Fig. 3. In the NaCl media, the PXRD pattern of residual particles was identical to that of the TFLX 1/2HCl reference material. In the Na_2_SO_4_ media, the PXRD pattern of residual particles was identical to that of the TFLX FF reference material.

**Fig. 4:**
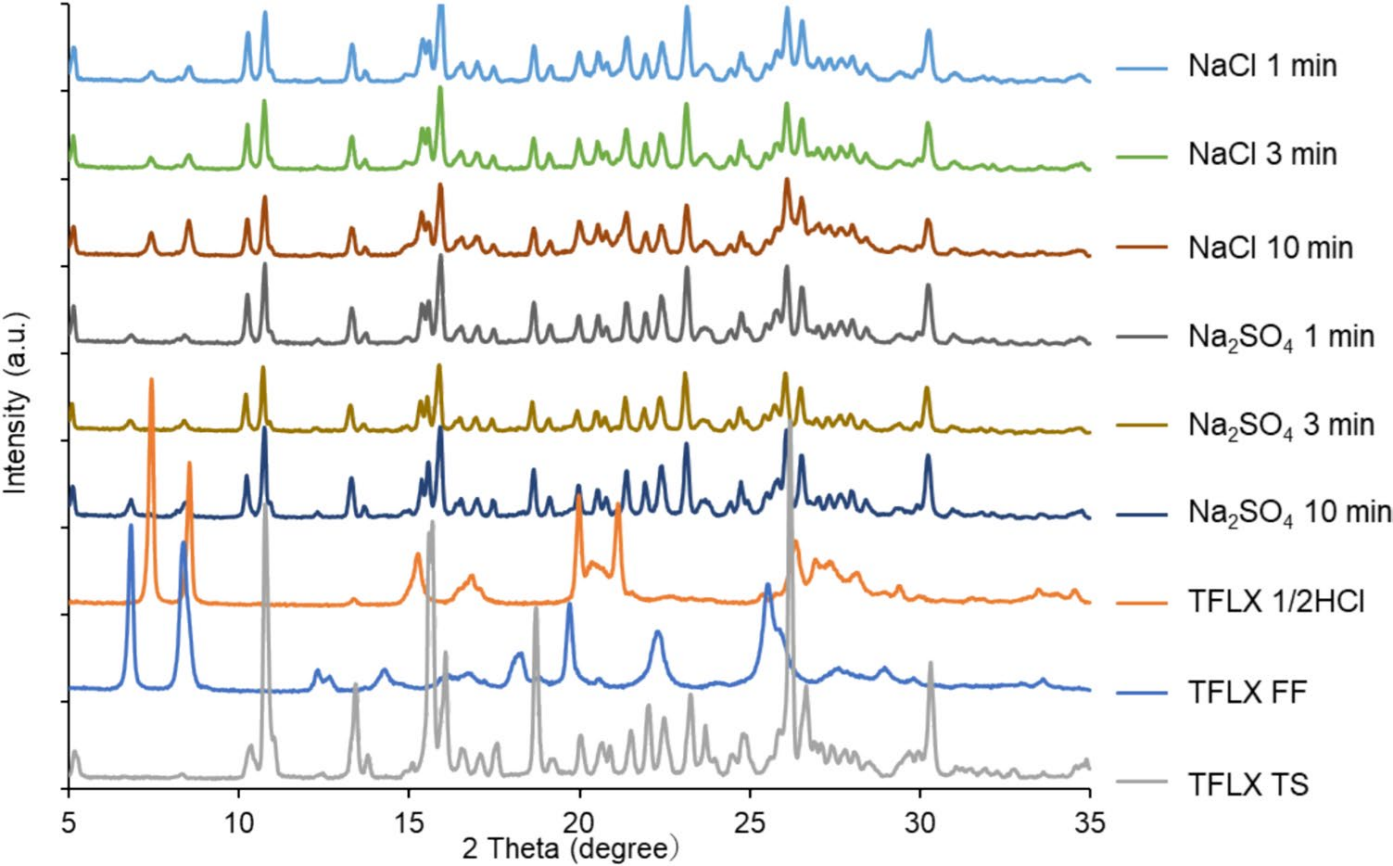
PXRD patterns of residual particles in dissolution tests at early time points in 10 mM bicarbonate buffer containing NaCl or Na_2_SO_4._

Figure 4 shows the time course of the PXRD patterns of the residual particles in 10 mM BCB. In the NaCl medium, the characteristic peaks of TFLX 1/2HCl appeared at early time points (< 1 min). In the Na_2_SO_4_ medium, the characteristic peaks of TFLX FF appeared at early time points (< 1 min).

### Surface Morphology Observation by Scanning Electron Microscopy

**Fig. 5:**
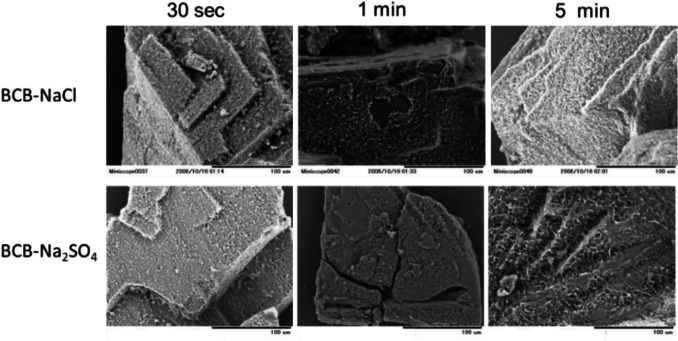
SEM images of the particles after mixing TFLX TS with pH 6.5 10 mM BCB buffer solutions containing NaCl or Na_2_SO_4_ (*I* = 0.14 M).

The SEM images of residual particles after mixing with BCB are shown in Fig. 5. Immediately after mixing with the media, fine needle-like crystals appeared on the crystal surface of TFLX TS.

### Real-time Polarized Light Microscopic Observation

**Fig. 6:**
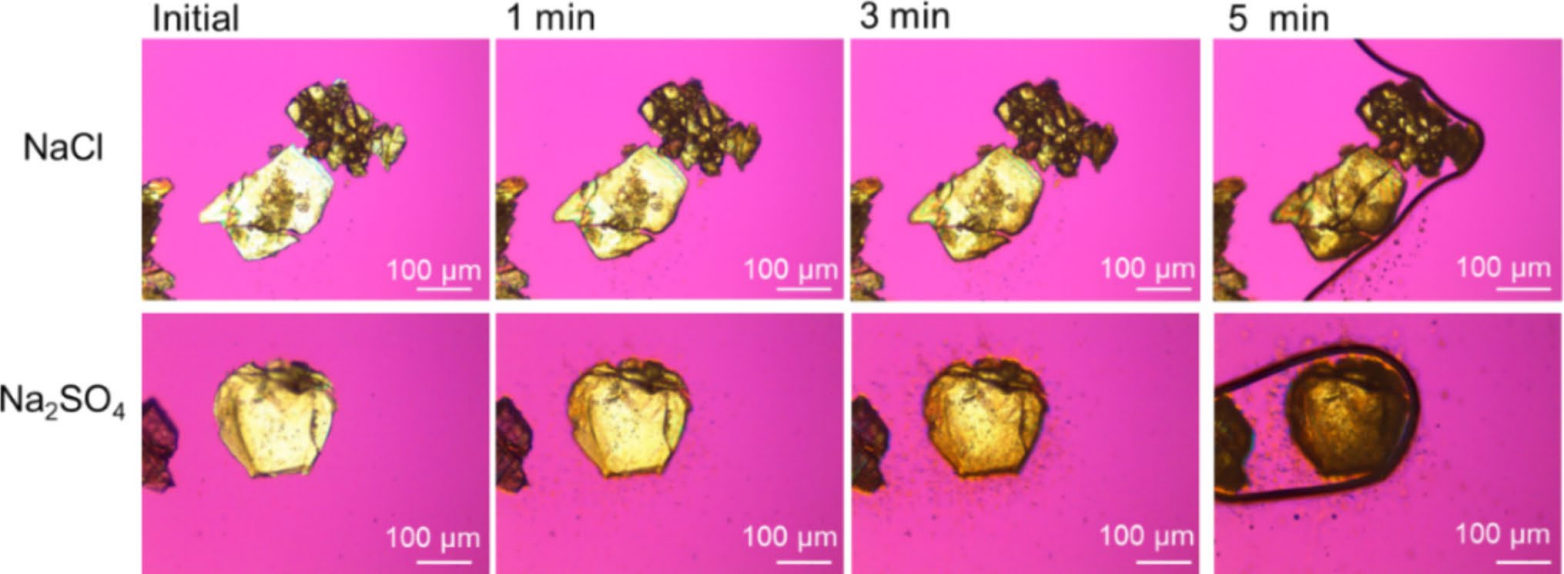
Real-time polarized light microscopic observation of TFLX TS in pH 6.5, 10 mM BCB with NaCl and Na_2_SO_4_ (*I* = 0.14 M).

In real-time polarized light microscopic observation, the dissolution patterns were different between the NaCl and Na_2_SO_4_ media (Fig. 6). In the Na_2_SO_4_ medium, the precipitation of crystals near the TFLX TS particles and the change of the surface texture of the TFLX TS particles were observed. On the other hand, only the change of surface texture was observed in the NaCl medium.

### pH Solubility Profile

**Fig. 7:**
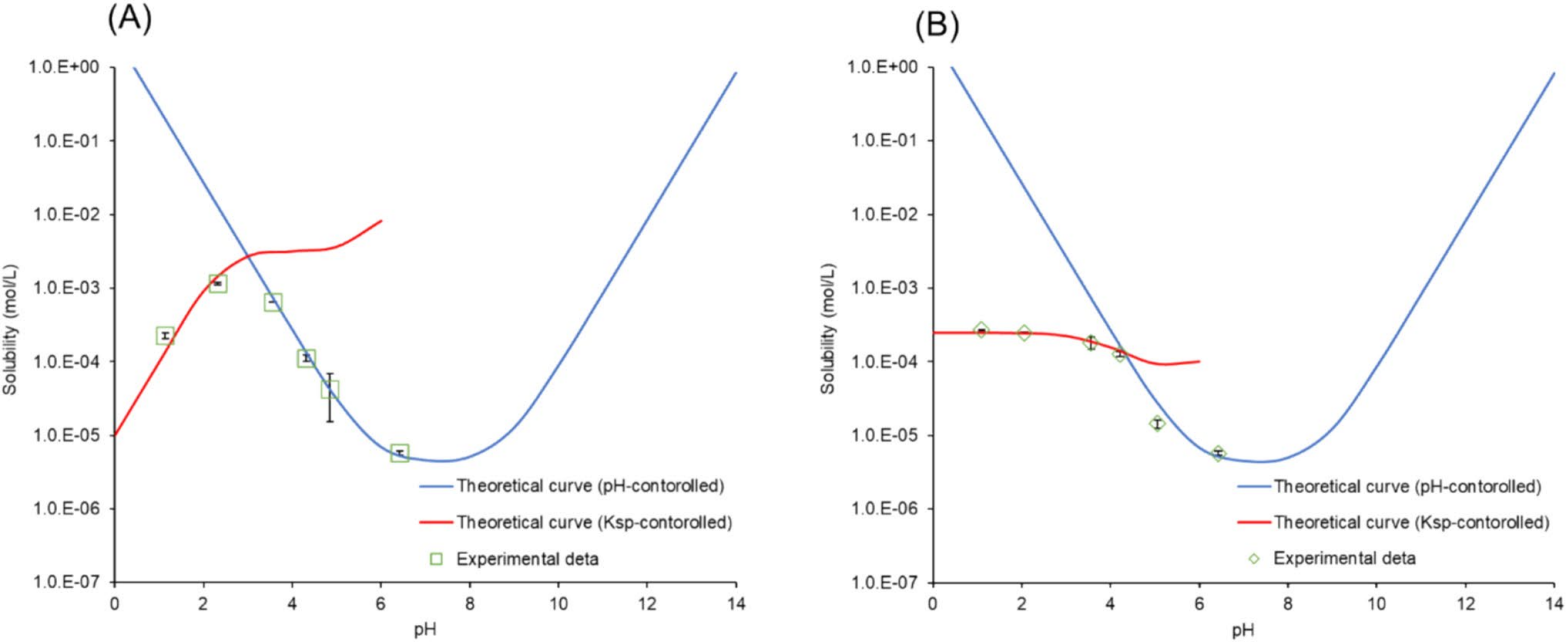
pH solubility profiles of TFLX. pH was adjusted by (**A**) p-toluenesulfonic acid or (**B**) HCl. The horizontal axis shows the final equilibrium pH value. The red and blue lines are the theoretical curves for the *K*_*sp*_ and pH-controlled regions, respectively. Mean ± S.D., N = 3.

The pH solubility profiles were measured starting with TFLX FF (Fig. 7). The pH value was adjusted by HCl or p-toluenesulfonic acid. When pH was adjusted by p-toluenesulfonic acid, the residual particles below pH 2.3 were identified as TFLX TS by PXRD (Supplementary Material Figure S5). When pH was adjusted by HCl, the residual particles below pH 3.4 were identified as TFLX 1/2HCl (Supplementary Material Figure S6). The solubility product (*K*_*sp*_) of TFLX TS (*K*_*sp,TFLX TS*_ = [TFLX∙H^+^][TS^−^]) was estimated to be 1.0 × 10^–5^ mol^2^/L^2^ from the pH solubility profile (Fig. 7A). In the case of TFLX 1/2HCl, *K*_*sp*_ is defined as *K*_*sp,TFLX 1/2HCl*_ = [TFLX∙H^+^][TFLX][Cl^−^]. The *K*_*sp,TFLX 1/2HCl*_ value was estimated to be 1.0 × 10^–13^ mol^3^/L^3^ from the pH solubility profile (Fig. 7B). The theoretical equations for the *K*_*sp*_ and pH-controlled regions are shown in the Supplementary Material.

## Discussion

### Effect of NaCl on the Dissolution Profile of Tosufloxacin Tosylate Monohydrate

The dissolution profiles of TFLX TS differed in the presence and absence of NaCl in both BCB and PPB. Because TFLX TS is a non-HCl salt, this phenomenon cannot be explained by the common ionic effect of the chloride ion (Cl⁻) directly suppressing the dissociation of a salt-form drug. This is an important case showing that the dissolution profile of a salt-form drug can be impacted by physiological NaCl at a neutral pH, even when the counter ion is neither Na⁺ nor Cl⁻.

The residual particles after the dissolution test were TFLX 1/2HCl in the NaCl media and TFLX FF in the Na_2_SO_4_ media. This explains the difference in the final bulk phase pH values between the NaCl and Na_2_SO_4_ media at low β (Table II). When TFLX was precipitated as a free form (zwitterionic form), p-toluenesulfonic acid was released in the solution, resulting in a decrease in the pH value. When TFLX 1/2HCl precipitated, the decrease in pH caused by the release of p-toluenesulfonic acid was partially offset by the removal of HCl, resulting in a smaller pH decrease. Because TFLX 1/2HCl is a complex of TFLX∙H^+^, TFLX, and Cl^−^, its precipitation releases two p-toluenesulfonic acids and removes one HCl into/from the solution.

### Particle Surface Precipitation or Bulk Phase Precipitation?

The results of SEM and RT-PLM studies, as well as the PXRD data of the residual particles at the early dissolution time point, indicated that TFLX 1/2HCl precipitated on the particle surface of TFLX TS in the NaCl media. In the previous bulk-phase pH-shift precipitation study [22], TFLX FF precipitated from the bulk-phase solution even in the presence of NaCl, further supporting that the precipitation of TFLX 1/2HCl occurred on the particle surface of TFLX TS in the dissolution test.

In addition, the dissolution profile of TFLX TS depended on the buffer species in the present study. In contrast, in the previous bulk phase pH-shift precipitation study [19], the precipitation of TFLX FF at pH 6.5 was little affected by buffer species. Furthermore, the precipitation induction time of TFLX FF is slow at 30 μg/mL or less at pH 6.5 [22]. Considering that the maximum TFLX concentration was less than 30 μg/mL in most cases of the present study, it is unlikely that rapid precipitation of TFLX FF occurred in the bulk phase. Together with the results of SEM and RT-PLM studies, as well as the PXRD data of the residual particles at the early dissolution time point, these results indicated that the precipitation of TFLX FF also occurred on the particle surface of TFLX TS in the Na_2_SO_4_ media.

### Effect of Buffer Species and Buffer Capacity on Particle Surface Precipitation

The dissolution profiles of TFLX TS in both the NaCl and Na_2_SO_4_ media were affected by buffer species and buffer capacity, suggesting that the particle surface precipitations of TFLX 1/2HCl and TFLX FF were affected by these factors.

In the case of the precipitation of TFLX FF, the effect of buffer species and buffer capacity can be explained by their effect on the surface pH [11, 19, 25, 29]. The rates of precipitation processes (both nucleation and particle growth) depend on the supersaturation ratio (SR) of TFLX FF against the intrinsic solubility (*S*_*0*_) (SR = [TFLX]_ss_/* S*_*0*_, where the subscript ss indicates the supersaturated concentration). At the surface of TFLX TS, the solution is in equilibrium with the solid of TFLX TS, so that the concentration of protonated TFLX becomes constant ([TFLX∙H^+^]ss = *K*_*sp,TFLX TS*_^*1/2*^). At pH <  < p*K*_*a2*_, the concentration of the anionic form is negligible. Therefore, the supersaturation ratio of TFLX FF (SR_TFLX FF_) is expressed as,

1 $${SR}_{TFLX FF}=\frac{{\left[TFLX\right]}_{ss}}{{S}_{0}}=\frac{{\left[TFLX\bullet {H}^{+}\right]}_{ss}}{{S}_{0}}\frac{{\left[TFLX\right]}_{ss}}{{\left[TFLX\bullet {H}^{+}\right]}_{ss}}=\frac{{{K}_{sp,TFLX TS}}^{1/2}}{{S}_{0}}\frac{{K}_{a1}}{[{H}^{+}]}$$

**Fig. 8:**
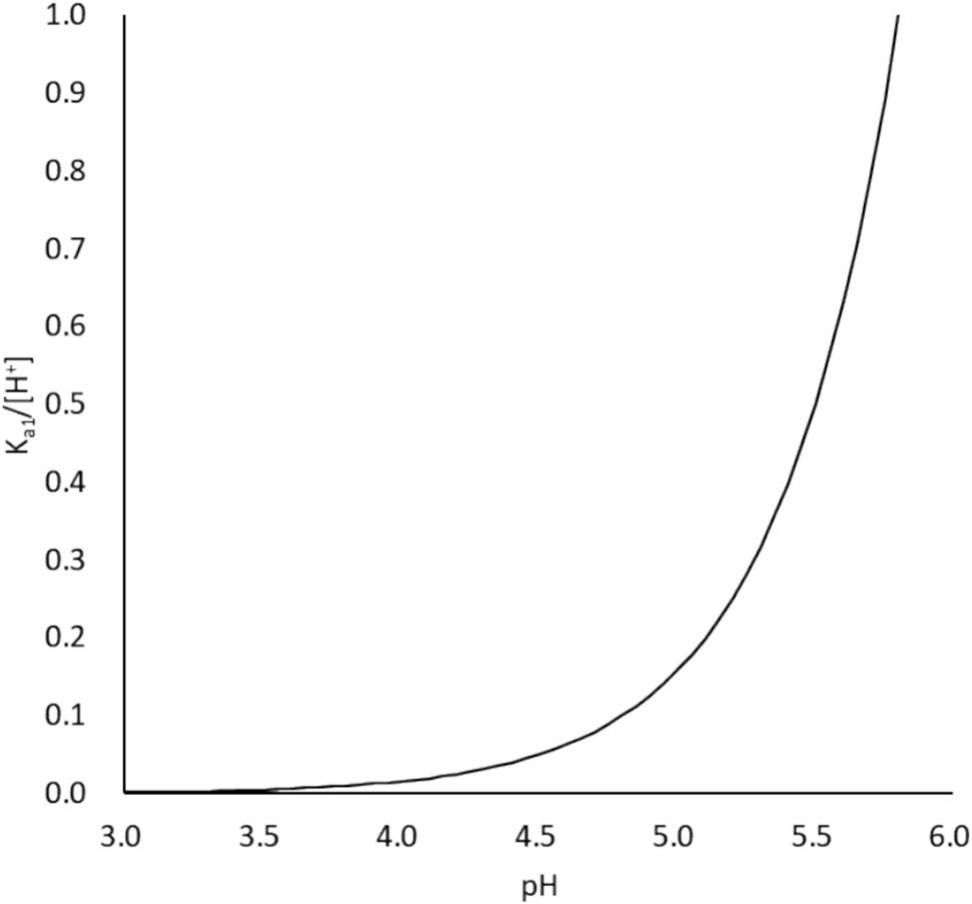
pH dependency of *K*_*a1*_/[H^+^].

*K*_*a1*_/[H^+^] and SR_TFLX FF_ steeply increase as pH increases (Fig. 8).

As discussed above, the precipitation of TFLX FF reduces the pH value. The surface pH neutralization rate of BCB is slower than that of PPB [4], due to the slow hydration/dehydration rate of CO_2_. Therefore, the surface pH becomes lower in BCB than in PPB. The lower the surface pH, the lower SR_TFLX FF_, and the less precipitation of TFLX FF. Similarly, as the buffer capacity becomes lower, the surface pH becomes lower, and the precipitation of TFLX FF becomes less. Therefore, the dissolution of TFLX TS becomes greater in BCB than in PPB and at lower β.

Similarly to the case of TFLX FF, the precipitation of TFLX 1/2HCl also reduces the pH value. The precipitation rate of TFLX 1/2HCl depends on the supersaturation ratio against the solubility product of TFLX 1/2HCl (SR _TFLX 1/2HCl_) (see SI for details).

2 $${SR}_{TFLX\, 1/2HCl}=\frac{{\left[TFLX\bullet {H}^{+}\right]}_{ss}{\left[TFLX\right]}_{ss}{\left[{Cl}^{-}\right]}_{ss}}{{K}_{sp,TFLX 1/2HCl}}=\frac{{K}_{sp,TFLX TS}}{{K}_{sp,TFLX 1/2HCl}}{\left[{Cl}^{-}\right]}_{ss}\frac{{K}_{a1}}{[{H}^{+}]}$$

Therefore, similarly to the case of TFLX FF, the precipitation of TFLX 1/2HCl becomes less in BCB than in PPB and at lower β.

### pH—Solubility Profile of a Hemi-Hydrochloride Salt

In this study, the hemi-hydrochloride salt of TFLX was formed in the NaCl media. In the literature, only a few hemi-hydrochloride salts of a drug have been reported [30–32]. To estimate the solubility product of TFLX 1/2HCl and TFLX TS, the pH-solubility profile of TFLX was measured with the pH adjustment by HCl or p-toluenesulfonic acid, respectively. The experimental data were in good agreement with the theoretical line (Fig. 7B), further supporting the stoichiometry of hemi-hydrochloride (*K*_*sp,TFLX1/2HCl*_ = [TFLXH^+^][TFLX][Cl^−^]). The low *K*_*sp*_ value of TFLX 1/2HCl is in good agreement with its rapid precipitation in the presence of NaCl on the particle surface of TFLX TS.

### Diversity of Particle Surface Precipitation Phenomena

In this study, TFLX 1/2 HCl was found to precipitate on the surface of TFLX TS particles in the NaCl media, whereas TFLX FF was found to precipitate in a Na_2_SO_4_ media. Recently, it was reported that the dissolution profiles of pioglitazone HCl and dantrolene Na were affected by buffer species and buffer capacity, due to the precipitation of their free-form crystals at the particle surface [11, 25]. In the case of prazosin HCl, the precipitation of a phosphate salt interfered with its dissolution in PPB, but not in BCB [33]. Liquid–liquid phase separation (LLPS) of free-form drug molecules can also occur at the particle surface of salt-form drugs [34, 35]. These results suggest that various types of precipitation phenomena can occur at the particle surface of a salt-form drug, affecting the dissolution profile. To improve dissolution testing and physiologically based biopharmaceutical modeling, a better understanding of the mechanisms of particle surface precipitation is required.

### Limitations of this Study

In this study, the effect of the other intestinal fluid constituents, such as bile micelles, on the dissolution profiles of TFLX TS was not investigated. Previously, the equilibrium solubility of TFLX was 1.6-fold higher in FaSSIF (3 mM taurocholic acid/0.75 mM egg lecithin) than in the blank FaSSIF without bile micelles [24]. However, bile micelles had little effect on the dissolution profiles of TFLX TS [24]. Further investigation is required to clarify the effect of bile micelles on the dissolution profiles of TFLX TS.

In conclusion, the poor dissolution profile of TFLX TS in biorelevant BCB containing NaCl was attributed to the particle surface precipitation of TFLX 1/2HCl. This study is an important case showing that the dissolution profile of a salt-form drug can be impacted by NaCl at pH 6.5, even when the counter ion is neither Na⁺ nor Cl⁻. This is a rare case showing that an HCl salt can precipitate in the NaCl media at neutral pH, despite the drug molecules mainly existing as a free form (zwitterion) in the bulk phase solution. The findings of this study would be important for a better understanding of the mechanisms of particle surface precipitation.

## Supplementary Information

Below is the link to the electronic supplementary material.

Supplementary file1 (DOCX 1087 KB)

## Data Availability

The datasets generated during and/or analysed during the current study are available from the corresponding author on request.
