# Supplementary material for: Dissolution Profile of Tosufloxacin Tosylate in Biorelevant Bicarbonate Buffer Containing Sodium Chloride: Precipitation of Hemi-hydrochloride Salt at the Particle Surface
Source: Pharm Res. 2025 Aug 11;42(8):1363–72. doi: 10.1007/s11095-025-03905-4 (PMC12405044; doi:10.1007/s11095-025-03905-4)
Supplement: Supplementary file 1 — Supplementary file1 (DOCX 1087 KB) [file 11095_2025_3905_MOESM1_ESM.docx]

Supplementary Material

Dissolution profile of tosufloxacin tosylate in biorelevant bicarbonate buffer containing sodium chloride: precipitation of hemi-hydrochloride salt at the particle surface

Nanami Okamoto, Hibiki Yamamoto, Kiyohiko Sugano^*^

Molecular Pharmaceutics Lab., College of Pharmaceutical Sciences, Ritsumeikan University, 1-1-1, Noji-higashi, Kusatsu, Shiga 525-8577, Japan

*Corresponding author. Tel.: +81-77-561-2773; E-mail address: suganok@fc.ritsumei.ac.jp (K. Sugano).

1. Preparation of sample and reference materials of TFLX salts


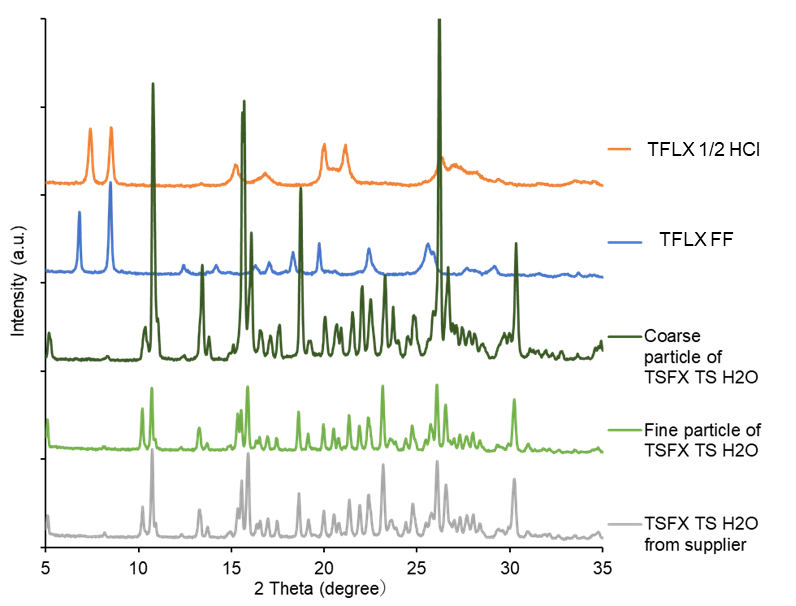


Figure S1 PXRD data of recrystallized materials


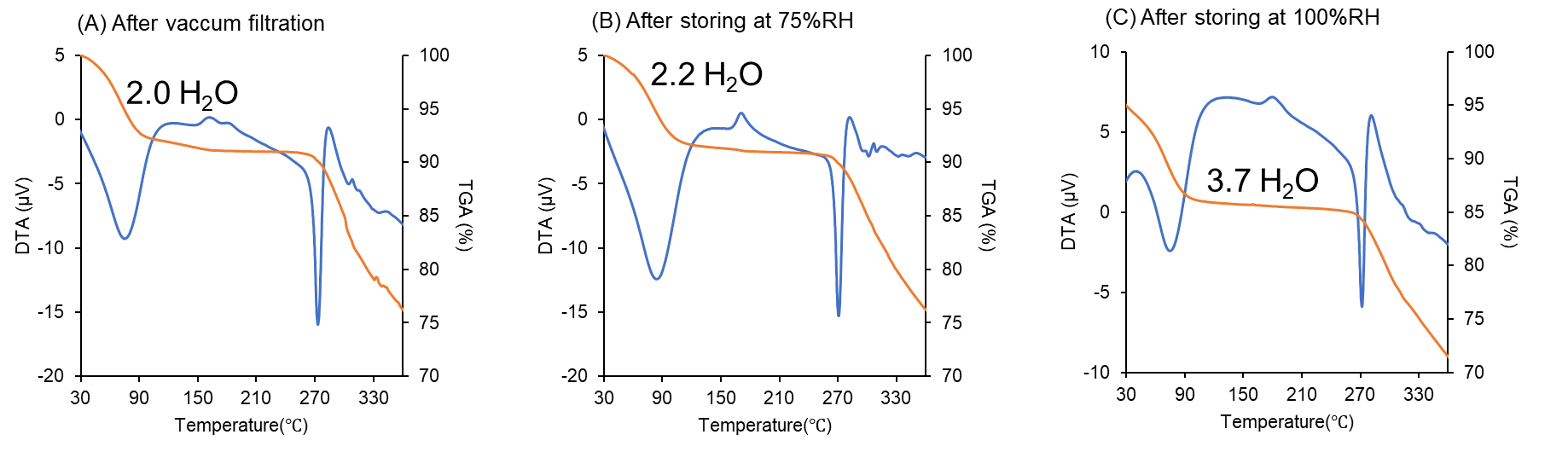


Figure S2 TG/DTA data of TFLX FF


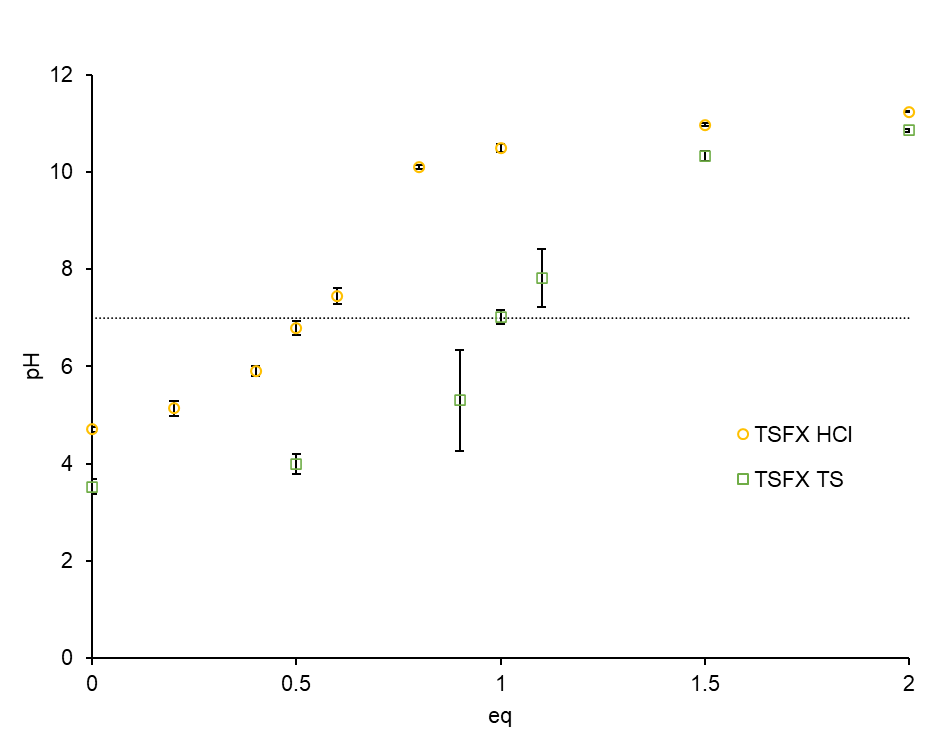


Figure S3 pH titration data of TFLX salts (p-toluenesulfonic acid (TS) and hydrochloride (HCl) salts). Each salt (10 mg) was suspended in distilled water (10 mL) at room temperature (25 ± 2 °C). NaOH (0.1 mol/L) was added in a stepwise manner. Mean ± S.D., N = 3.


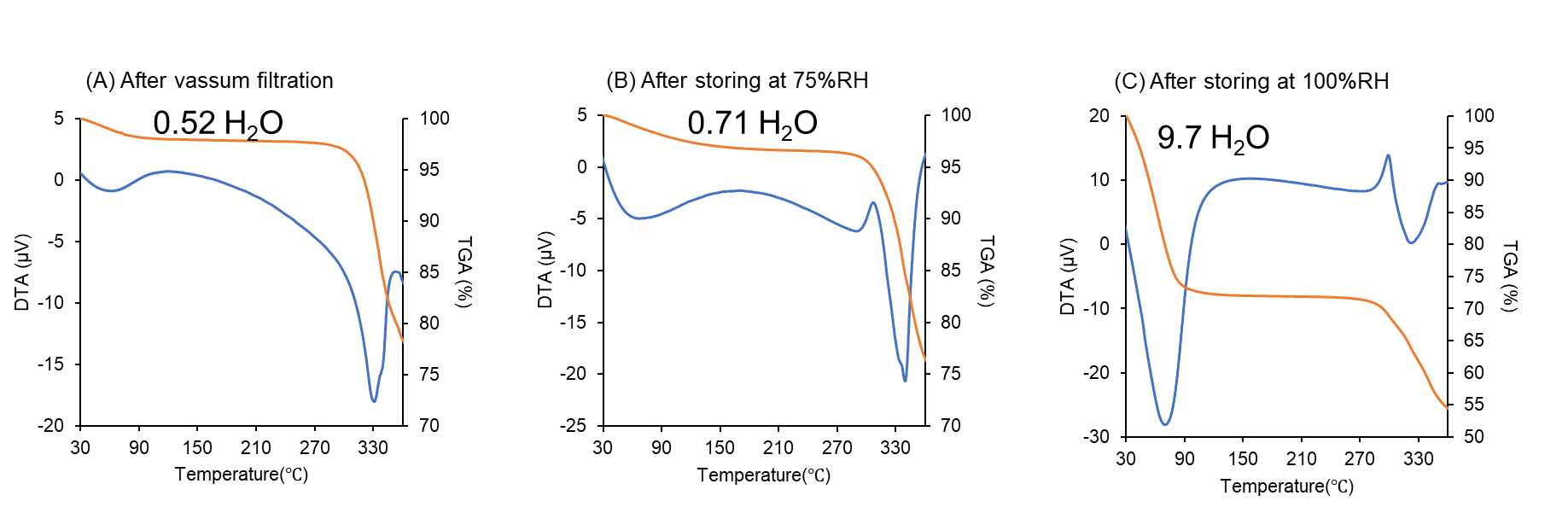


Figure S4 TG/DTA data of TFLX 1/2HCl

1. Residual particles in pH solubility measurements


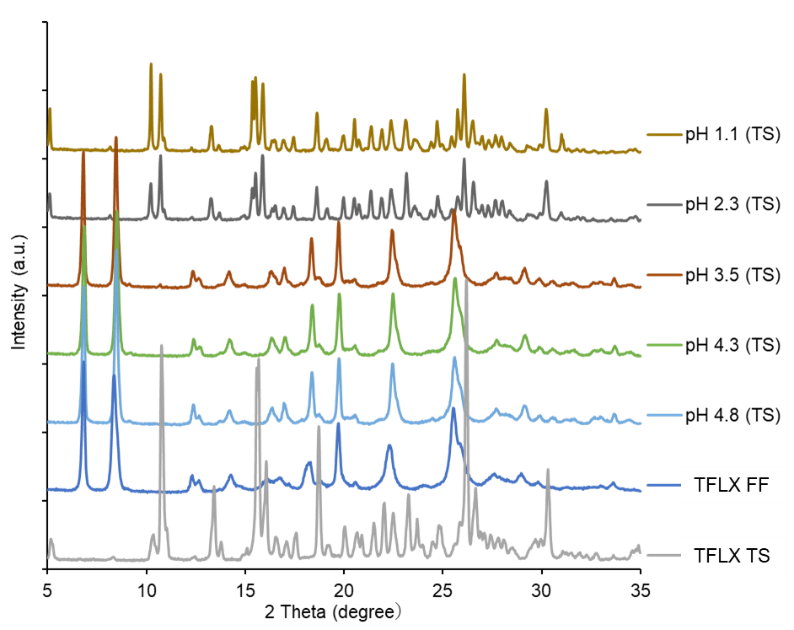


Figure S5 PXRD data of residual particles in pH solubility profile measurement (pH adjusted by p-toluenesulphonic acid).


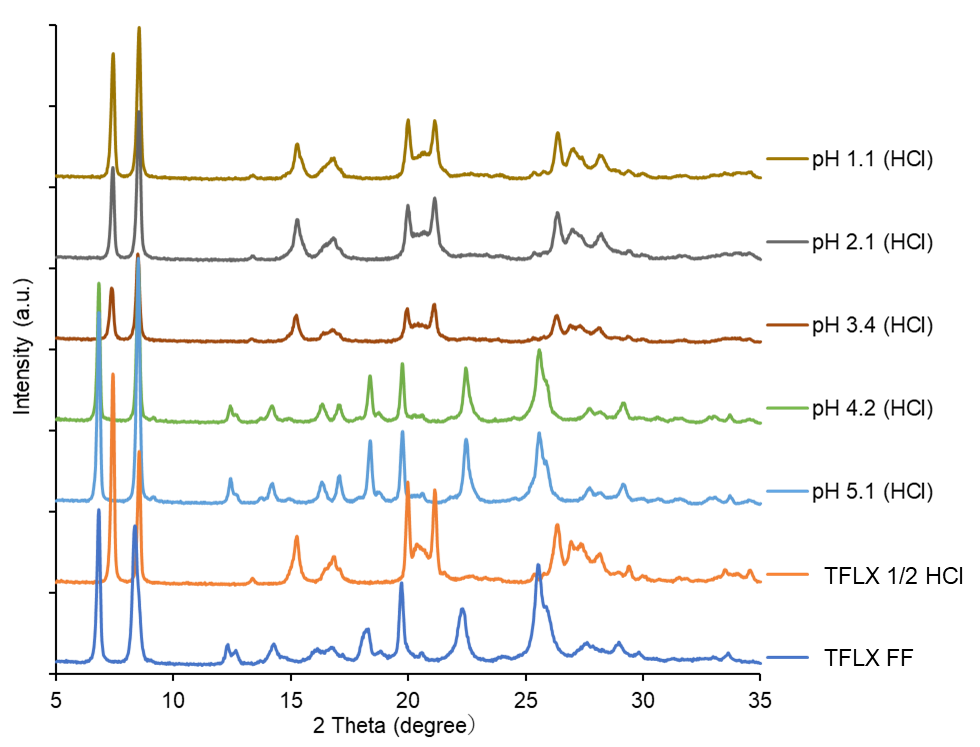


Figure S6 PXRD data of residual particles in pH solubility profile measurement (pH adjusted by HCl).

1. Theoretical equations of pH solubility profiles
   1. pH-controlled region

In the pH-controlled region, the equilibrium maker (residual solid) is the free form TFLX (zwitterion) (DH).

| $K_{a1}=\frac{\left[ DH \right]\left[ H^{+} \right]}{\left[ DH\cdot H^{+} \right]}={10}^{-5.8}$ | (S1) |
| --- | --- |
| $K_{a2}=\frac{\left[ D^{-} \right]\left[ H^{+} \right]}{\left[ DH \right]}={10}^{-8.7}$ | (S2) |

The charge-neutral fraction (f_0,±1_) is

| $f_{0,\pm1}=\frac{\left[ DH \right]}{\left[ D^{-} \right]+\left[ DH \right]+\left[ DH\cdot H^{+} \right]}=\frac{1}{\frac{\left[ D^{-} \right]}{\left[ DH \right]}+1+\frac{\left[ DH\cdot H^{+} \right]}{\left[ DH \right]}}=\frac{1}{\frac{\left[ H^{+} \right]}{K_{a1}}+1+\frac{K_{a2}}{\left[ H^{+} \right]}}$ | (S3) |
| --- | --- |

The equilibrium solubility at a pH (*S_eq_*) is,

| $S_{eq}=S_{0}/f_{0,\pm1}$ | (S4) |
| --- | --- |

- 1. *K_sp_*-controlled region
     1. Mono-salt

The solubility product (*K_sp_*) of a mono-salt with an acid (HA) is defined as,

| $K_{sp}=\left[ DH\cdot H^{+} \right]\left[ A^{-} \right]$ | (S5) |
| --- | --- |

At pH << p*K_a2_*, the concentration of the anionic form ($\left[ D^{-} \right]$) is negligible. Therefore, only DH and DH∙H^+^ are considered as the molecular species of a drug. The charge neutrality is

| $\left[ H^{+} \right]+\left[ DH\cdot H^{+} \right]=\left[ {OH}^{-} \right]+\left[ A^{-} \right]$ | (S6) |
| --- | --- |

Using *K_w_* = [H+][OH-] and rearranging,

| $\left[ A^{-} \right]=\left[ H^{+} \right]+\left[ DH\cdot H^{+} \right]-\frac{K_{w}}{\left[ H^{+} \right]}$ | (S7) |
| --- | --- |

By inserting Eq. S7 into Eq. S5,

| $K_{sp}=\left[ DH\cdot H^{+} \right]\left( \left[ H^{+} \right]+\left[ DH\cdot H^{+} \right]-\frac{K_{w}}{\left[ H^{+} \right]} \right)$ | (S8) |
| --- | --- |

Therefore,

| $K_{sp}-\left[ DH\cdot H^{+} \right]\left( \left[ H^{+} \right]+\left[ DH\cdot H^{+} \right]-\frac{K_{w}}{\left[ H^{+} \right]} \right)=0$ | (S9) |
| --- | --- |

By solving this quadratic equation, [DH∙H^+^] can be calculated. *S_eq_* at pH << p*K_a2_* can be calculated as

| $S_{eq}=\left[ DH\cdot H^{+} \right]+\left[ DH \right]=\left[ DH\cdot H^{+} \right]/\left( 1-f_{0,\pm1} \right)$ | (S10) |
| --- | --- |

- - 1. Hemi-salt

The solubility product of a hemi-salt is defined as,

| $K_{sp}=\left[ DH\cdot H^{+} \right]\left[ DH \right]\left[ A^{-} \right]$ | (S11) |
| --- | --- |

Similarly,

| $K_{sp}-\left[ DH\cdot H^{+} \right]\frac{{\left[ DH\cdot H^{+} \right]K}_{a1}}{\left[ H^{+} \right]}\left( \left[ H^{+} \right]+\left[ DH\cdot H^{+} \right]-\frac{K_{w}}{\left[ H^{+} \right]} \right)=0$ | (S12) |
| --- | --- |

By solving this cubic equation, [DH∙H^+^] can be calculated. In this study, the bisection method was used to seek the root of this equation. *S_eq_* can be calculated by Eq. S10.

1. Theoretical equations of the supersaturation ratio of TFLX 1/2HCl (D 1/2HCl)

At pH << p*K_a2_*, the concentration of the anionic form ($\left[ D^{-} \right]$) is negligible. Therefore, only DH and DH∙H^+^ are considered as the molecular species of a drug. When the system is in equilibrium with the mono-salt of a drug with an acid (HA_I_) (*K_sp,AIsalt_*, DH∙H^+^: A_I_^-^ = 1:1), the supersaturation ratio against the hemi-salt of the drug with another acid (HA_II_) (*K_sp,AIIsalt_*, DH∙H^+^: DH: A_II_^-^ = 1: 1: 1) is expressed as,

| $SR=\frac{\left[ DH\cdot H^{+} \right]_{ss}\left[ DH \right]_{ss}\left[ {Cl}^{-} \right]_{ss}}{K_{sp,A_{II}salt}}=\frac{{\left[ DH\cdot H^{+} \right]_{ss}}^{2}\left[ {Cl}^{-} \right]_{ss}}{K_{sp,A_{II}salt}}\frac{\left[ DH \right]_{ss}}{\left[ DH\cdot H^{+} \right]_{ss}}$ | (S13) |
| --- | --- |

where the subscript ss indicates a supersaturated state. Since the equilibrium maker is a 1:1 salt,

| ${\left[ DH\cdot H^{+} \right]_{ss}}^{2}=K_{sp,K_{sp,A_{I}salt}}$ | (S14) |
| --- | --- |

By definition, *K_a1_*/[H^+^] = [DH]/[DH∙H^+^]. Therefore,

| $SR=\frac{K_{sp,K_{sp,A_{I}salt}}}{K_{sp,K_{sp,A_{II}salt}}}\left[ {Cl}^{-} \right]_{ss}\frac{K_{a1}}{[H^{+}]}$ | (S15) |
| --- | --- |
